# Association of smoking or tobacco use with ear diseases among men: a retrospective study

**DOI:** 10.1186/1617-9625-10-4

**Published:** 2012-04-03

**Authors:** Kiran Gaur, Neeraj Kasliwal, Rajeev Gupta

**Affiliations:** 1 Department of Statistics, Banasthali University, Jaipur 302001, India; 2 Dr KC Kasliwal's ENT Centre, Paanch Batti, Jaipur 302001, India; 3 17, Luv -Kush Nagar I, Tonk Phatak, Jaipur 302015, India

## Abstract

**Background:**

Health related behaviour specially smoking and tobacco in any form are major determinants of health and lead to health inequities. Tobacco leads to various health problems including ear, nose and throat diseases.

**Objective:**

To determine the influence of smoking or tobacco use on ear diseases we performed a retrospective study among men.

**Method:**

Of 11454 subjects of different age-groups there were 4143 men aged 20-60 years who were evaluated for demographic variables, smoking/tobacco use and middle and internal ear diseases. Descriptive statistics and age adjusted logistic regression analyses were performed.

**Results:**

Among the 4143 men, 1739 (42.0%) were smokers or used tobacco. In smokers/tobacco users compared to non-users the age adjusted odds ratios and 95% confidence intervals (CI) for chronic suppurative otitis media were 1.13 (CI 0.96-1.34), acute otitis media 1.16 (CI 0.82-1.64), suppurative otitis media 1.21 (CI 0.79-1.84), otosclerosis 0.97 (CI 0.52-1.33) (p > 0.05) and for overall middle ear diseases was 1.15 (CI 0.99-1.33, p = 0.05). For internal ear diseases the age adjusted odds ratios were for sensorineural hearing loss 1.12 (CI 0.92-1.58), 0.12 (CI 0.42-0.93) for vertigo and tinnitus and overall internal ear diseases were 0.97 (CI 0.77-1.22, p = 0.81). Among men 40-60 years there was a significantly greater risk for both middle ear (OR 1.73, CI 1.29-2.30) and internal ear diseases (OR 1.94, CI 1.24-3.04) (p < 0.001).

**Conclusion:**

Smoking/tobacco use is significantly associated with greater prevalence of middle and internal ear diseases among middle-aged men in India.

## Introduction

The hazardous effect of tobacco on health is universally known[1]. The tobacco-induced negative effects on health include various types of cancers, heart disease, strokes, emphysema, and a slew of other serious (and often fatal) illnesses as observed in epidemiological and clinical studies[1,2]. Evidences have accumulated in recent years on the adverse effects of smoking and tobacco use on ear diseases and hearing among different population groups[3-10]. Some studies, on the other hand, have reported absence of such an association[11-13]. All forms of smoking and tobacco use are harmful[1,14,15] and there are significant data on relationship of smoking and middle-ear diseases in children (by passive smoking) and in adults[3,4]. Among adults the risks of internal ear diseases especially hearing loss also increases with smoking[5,9]. The risk of becoming hearing-impaired often increases with the number of cigarettes smoked, as well as with the intensity and the duration of exposure to smoke and tobacco[10]. Relationship of non-smoked tobacco use and combined smoking and tobacco use has not been well studied.[14,15]

Previous studies have mainly focused on the independent association of smoking on ear diseases and hearing but almost none have evaluated combined effects of smoking and non-smoked tobacco use on different type of ear diseases and hearing[3-15]. We previously reported results of a retrospective study of 30 year trends in various otolaryngorhinological diseases in India[16]. In this study we also collected data on smoking and tobacco use and the present study was undertaken to evaluate the association between these habits and middle and internal ear disease in men. Number of female tobacco users was low. The study also aimed to evaluate the role of smoking/tobacco use at different age-groups as it is likely that more prolonged exposure shall lead to greater ear diseases.

## Methods

The study was conducted at a non-government ENT center at Jaipur. From 1975 to 2005, > 125,000 patients of different diseases were evaluated here. We randomly selected patients (n = 11,454) at an interval of five years to evaluate disease trends.^16 ^For the purpose of the present study we evaluated a sample of 4143 males (as female tobacco users were small in number) aged 20-60 years after exclusion of children where tobacco use or smoking was less. Men who gave history of tobacco smoking (cigarettes, bidis and others) or used non-smoked tobacco (chewing, snuff, etc.) were considered as tobacco users. Among the smokers/tobacco users were about 50%, isolated tobacco chewing was present in 20% and the rest both smoked and chewed tobacco. Demographic details were available in the medical records but other details including details of other addictions and risk factors were not available. All the case-files were screened and coded for diagnosis by a senior clinician. The patients were classified according to complaints and clinical examination into major diagnostic groups and disease conditions according to International Classification of Diseases (ICD-9) as reported earlier[16]. The diseases were broadly classified into middle ear and internal ear diseases. The study is approved by ethical committee of Monilek Hospital and Research Centre, Jaipur.

### Statistical analyses

All the data were computerized. Statistical analyses were performed using SPSS program, version 12.0 (SPSS Inc, Chicago). Descriptive statistics are presented. Patients were classified according to their habit of tobacco intake (independent variable, dichotomous) and disease status (dependent variable). Odds ratios and 95% confidence intervals (CI) were determined using logistic regression analysis. It was a generalized linear model used for binomial regression. Like other forms of regression analysis, it makes use of one or more predictor variables that may be either numerical or categorical. Tobacco intake was considered as independent variable and various middle and internal ear diseases were treated as dependent variable.

P values < 0.05 were considered significant.

## Results

In the 11,454 study subjects there were 4143 men aged 20-60 years. Among these 1739 (42.0%) were smokers or used tobacco. Of these 1054 (60.6%) belonged to age-group 20-39 years and 685 (39.4%) were aged 40-60 years. Tobacco non-users were 2404 and 1751 (72.8%) were aged 20-39 years and 653 (27.2%) were in age-group 40-60 years. Eight hundred and sixty seven (49.8%) tobacco users and 1438 (59.8%) non-users were residents of urban area and 872 (50.2%) tobacco users and 966 (40.2%) non-users were the residents of semi urban and rural areas.

Among the middle ear diseases chronic suppurative otitis media (CSOM), acute otitis media (AOM), secretory otitis media (SOM) and otosclerosis were found to be the most common with 659 (15.9%), 136 (3.2%), 88 (2.1%) and 41 (0.9%) patients respectively when adjusted for age. Amongst tobacco users 294 (16.9%) had CSOM, 62 (3.5%) had AOM, 41 (2.3%) had SOM and 17 (0.9%) had otosclerosis. The age adjusted odds ratio and 95% CI for tobacco users for CSOM was 1.13 (CI 0.96-1.34), AOM 1.16 (CI 0.82-1.64), SOM 1.21 (CI 0.79-1.84) and for otosclerosis it was 0.97 (CI 0.52-1.82) (*p *> 0.05) (Table 1). The middle ear diseases were present in 920 subjects of which 411 (23.6%) were tobacco users with odds ratio was 1.15 (CI 0.99-1.33 *p *= 0.05) (Table 2). For internal ear diseases sensorineural hearing loss (SNHL) and vertigo/tinnitus were the most common diseases. SNHL was observed in 222 (5.3%) patients and among tobacco users were in 103 (5.9%) men. Vertigo and tinnitus was observed in 117 (2.8%) subjects and among tobacco users were in 37 (2.1%). The age adjusted odds ratio was 1.12 (CI 0.92-1.58) for SNHL and 0.12 (CI 0.42-0.93) for vertigo and tinnitus (Table 1). For internal ear disease as whole, we had a sample of 336 males and tobacco use was in 139 (7.9%). The odds ratio for internal ear disease among tobacco users was 0.97 (0.77-1.22, *p *= 0.81) (Table 2).

**Table 1:** The effect of any tobacco use on the development of middle and internal ear

Disease		Total (n = 4143)	Smoking/Tobacco use (n = 1739)	Non-user (n = 2404)	Odds Ratio (95% CI)	P value
Middle Ear Disease	Chronic suppurative otitis media	659 (15.9)	294 (16.9)	365 (15.1)	1.13 (0.96-1.34)	0.13
	
	Acute otitis media	136 (3.2)	62 (3.5)	74 (3.0)	1.16 (0.82-1.64)	0.38
	
	Secretory otitis media	88 (2.1)	41 (2.3)	47 (1.9)	1.21 (0.79-1.84)	0.37
	
	Otosclerosis	41 (0.9)	17 (0.9)	24 (1.0)	0.97 (0.52-1.82)	1.0
Internal Ear Disease	Sensorineural hearing loss	222 (5.3)	103 (5.9)	119 (4.9)	1.12 (0.92-1.58)	0.16
	
	Vertigo & tinnitus	117 (2.8)	37 (2.1)	80 (3.3)	0.12 (0.42-0.93)	0.02

All the above values are adjusted for age.

Numbers in parentheses are percent unless specified.

*CI *confidence intervals

**Table 2:** Age-group specific odds-ratio for smoking/tobacco use and ear diseases

Disease	Age-Group	Smoking/tobacco use (n = 1739)	Non tobacco user(n = 2404)	Odds ratio (95% CI)	P value
Middle Ear	20-39	267(15.3)	394(16.4)	0.57(0.43-0.77)	0.0

	40-60	144(8.3)	23(5.1)	1.73(1.29-2.30)	0.0
	
	Total (Unadjusted)	411(23.6)	517(21.5)	1.13(0.97-1.31)	0.1
	
	Total (Age adjusted)	411 (23.6)	509 (21.1)	1.15 (0.99-1.33)	0.05

Internal Ear	20-39	50(2.9)	91(3.8)	0.51(0.33-0.81)	0.0

	40-60	96(5.5)	90(3.7)	1.94(1.24-3.04)	0.0
	
	Total (Unadjusted)	146(8.4)	181(7.5)	1.12(0.90-1.41)	0.3
	
	Total (Age adjusted)	139 (7.9)	197 (8.2)	0.97 (0.77-1.22)	0.81

Numbers in parentheses are percent unless specified; *CI *confidence intervals

Age specific odds ratios were also calculated to see the effect of age on middle and internal ear diseases. Among middle aged men 40-60 years there was a significantly greater risk for both middle ear (OR 1.73, CI 1.29-2.30) and internal ear diseases (OR 1.94, CI 1.24-3.04) (*p *< 0.001) (Table 2).

## Discussion

This study reveals that middle-aged tobacco user men had a significantly greater risk for both middle ear and internal ear diseases as compared to those who did not use tobacco. This difference is mainly due to greater prevalence of chronic middle ear diseases and sensorineural hearing loss in this group.

Cruickshanks et al.[5] performed a population based cross sectional study among 3753 subjects (43% men) in USA to determine association of hearing loss with smoking. There were 54% smokers and ex-smokers in this group with 40% smokers reporting > 10 pack years of smoking. Otological examination and pure-tone audiometery was performed and after adjusting for other risk factors such as age and sex, current smokers were 1.69 times as likely to have hearing loss as compared to non-smokers (CI 1.31-2.17). The present study shows lower odds ratio of 1.40 (CI 1.07-1.85) which could be due to the lower mean age in the present study. On the other hand, in the age-group corresponding to the US study odds for internal ear diseases are similar (1.94, CI 1.24-3.04). For middle ear diseases there are very limited data among adults and studies are mainly confined in children that have evaluated harmful effects of second-hand smoke[3,4,17]. Adair-Bischoff et al.[17] reported odds of 1.85 (CI 1.15-2.97) for children exposed to second-hand smoke by > 2 members of the household. We did not study children and therefore the results are not available. It is also likely that low odds of middle ear diseases (overall odds 1.13, CI 0.97 -1.31) as compared to sensorineural hearing loss (overall odds 1.40, CI 1.07-1.85) are due to confounding effects of different type of tobacco usage in the study. Larger studies with better classification of tobacco use are required to confirm the findings of the present study.

Strengths of the study include uniqueness (as this is the only large study from India), representativeness (registry data in an urban clinical practice) and a large sample size. Hearing loss is the most important otological disease related to smoking/tobacco use[1,5] and we also evaluated sensorineural hearing loss using the most reliable technique, pure-tone audiometery, and this is a study strength. We did not study the effects of smoking or non-smoked tobacco use separately as the information related to specific type of tobacco use was not available for these subgroups. Other limitations of this study include retrospective nature and lack of detailed description of amount of tobacco use. We also did not inquire regarding multiple confounders such as exposure to environmental noise, use of betel-quid, pan-masala, alcohol use and other addictions which all can independently influence the middle and internal ear diseases[8-11,15]. We did not also study the influence of tobacco use in women and young children as the absolute numbers were low.

## Conclusion

In conclusion, this study shows a significant association of smoking/tobacco use with hearing loss in middle-aged adults and adds to the growing list of diseases attributable to smoking and tobacco use[1]. Smoking and tobacco habit is taking its toll in this country and it has been predicted that in this century it would lead to more than a hundred million premature deaths in the country[18]. National level policy change[19] leading to total cessation of this habit is urgently required.

## Competing interests

All the authors have no competing interests relevant to this article.

## Authors' contributions

KG conceived, designed and implemented the study, provided statistical inputs and wrote the first draft of the manuscript. NK overviewed data collection and checked minor medical details about diseases. RG jointly conceived and designed the study, provided critical academic inputs and wrote the subsequent revision of the manuscript and prepared the final manuscript. All authors read and approved the final manuscript.
